# Jottings

**Published:** 1910-01-29

**Authors:** 


### Jottings.

The King has sent a present of linen to the Chelsea Hospital for Women.

The Sheppey Guardians invite the supply of a suitable horse ambulance for the conveyance of infectious cases and general use.

Sir S. Crossley will preside at the annual dinner of the Hospital Saturday Fund at the Holborn Restaurant on February 12.

The first meeting of the reconstituted board of the Hospital Saturday Fund for the current year will be held on February 19.

A dentist has been appointed at a salary of £50 a year to attend to the teeth of the children in the Mile End Guardians' Scattered Homes.

Mr. J. H. Levy, who was for some time chairman of the board of management of the Anti-Vivisection Hospital, has severed his connection with that institution.

The North Bierley Guardians are arranging that ratepayers in the Union may inspect the workhouse premises in view of the erroneous impressions created in the public mind through the publication of the Poor Law Commission Reports.

Hornsey Cottage Hospital was opened on Thursday, January 27, by the wife of the hon. treasurer, Mr. J. G. Abraham. The long delay is now over, and the people of Hornsey will have an institution worthy of its 100,000 inhabitants.

Coleraine Cottage Hospital, Londonderry, Ireland, at its recent annual meeting was able to report a legacy of £20 received from a former patient of the hospital. In the course of the year the operating-room has been enlarged and an economy of almost £100 has been effected.

The Local Government Board is announced to have sanctioned a proposal to convert the smallpox hospital at Morton Banks into a sanatorium for consumptives, thorough disinfection being made, at the meeting of the Keighley and Bingley Joint Hospital Board on January 19.

The Ecclesall Bierlow Guardians have passed a resolution in favour of a monthly return of surgical operations undergone at the Workhouse Infirmary, showing the names and ages of the inmates operated upon, the date, nature, and result of each operation, and the condition of the inmate on the date of the return.

The Bristol Dispensary, Castle Green, held its annual meeting on January 14. Mr. Fenwick Richards, hon. treasurer, said that the demand for medical relief had increased during the year, 12,388 being treated. Subscribers had bought many more books, the midwifery notes being in great demand. In spite of a deficit of £22 on their income, £130 14s. 7d. was still at the bank.

Among the principal grants made by the Hospital Saturday Fund on January 22 were :—London Hospital, £1,164; Middlesex, £471; St. Mary's, £457; University College, £448; St. George's, £445; Seamen's, £365; Westminster, £353; Guy's, £348; Royal Free, £341; King's College, £347; Great Northern, £334; Charing Cross, £333; West London, £326; German, £224; St. Thomas's, £200; Homœopathic, £154; Battersea General, £80; Cancer (Brompton), £210; Middlesex Cancer Wards, £158; East London (Children), £230; Hospital for Sick Children, £330; Queen's (Children), £269; Queen Charlotte's Lying-in, £165; Royal London Ophthalmic, £259; Royal National Orthopædic, £158; Samaritan Free (Women), £152; Chelsea (Women), £144. The total receipts of the Saturday Fund for the year reached £30,708, which was an increase upon 1908 of £878; and the awards were £1,338 more than in the previous year, five additional medical charities benefiting.

